# Potential of Peptides as Inhibitors and Mimotopes: Selection of Carbohydrate-Mimetic Peptides from Phage Display Libraries

**DOI:** 10.1155/2012/740982

**Published:** 2012-10-10

**Authors:** Teruhiko Matsubara

**Affiliations:** Department of Biosciences and Informatics, Faculty of Science and Technology, Keio University, 3-14-1 Hiyoshi, Kouhoku, Yokohama 223-8522, Japan

## Abstract

Glycoconjugates play various roles in biological processes. In particular, oligosaccharides on the surface of animal cells are involved in virus infection and cell-cell communication. Inhibitors of carbohydrate-protein interactions are potential antiviral drugs. Several anti-influenza drugs such as oseltamivir and zanamivir are derivatives of sialic acid, which inhibits neuraminidase. However, it is very difficult to prepare a diverse range of sugar derivatives by chemical synthesis or by the isolation of natural products. In addition, the pathogenic capsular polysaccharides of bacteria are carbohydrate antigens, for which a safe and efficacious method of vaccination is required. Phage-display technology has been improved to enable the identification of peptides that bind to carbohydrate-binding proteins, such as lectins and antibodies, from a large repertoire of peptide sequences. These peptides are known as “carbohydrate-mimetic peptides (CMPs)” because they mimic carbohydrate structures. Compared to carbohydrate derivatives, it is easy to prepare mono- and multivalent peptides and then to modify them to create various derivatives. Such mimetic peptides are available as peptide inhibitors of carbohydrate-protein interactions and peptide mimotopes that are conjugated with adjuvant for vaccination.

## 1. Introduction

A variety of glycoconjugate carbohydrate structures on the cell surface are important for biological events [1]. Carbohydrate structures on the cell surface change according to cell status, for example, during development, differentiation, and malignant alteration. Several glycoconjugates, including stage-specific embryonic antigen (SSEA)-3, SSEA-4, and tumor-rejection antigen (TRA)-1-60, are used as molecular makers of pluripotency to control the quality of induced pluripotent stem (iPS) cells [2]. Carbohydrate-protein interactions are the first cell surface events in cell-cell communication, following which processes such as infection and signal transduction occur. However, the reasons for the changes in carbohydrate structures on the cell surface are not clear. In addition, most receptors for glycoconjugates have not been identified. To investigate the biological roles of carbohydrates, sets of carbohydrates and their corresponding carbohydrate-binding proteins are required.

Carbohydrate-binding proteins such as plant lectins, bacterial toxins, and anticarbohydrate antibodies are available for studying carbohydrate-protein interactions [3, 4]. However, the repertoire of carbohydrate structures recognized by these proteins is limited and insufficient to cover the majority of structures. In addition, because carbohydrates are ubiquitous components of cell membranes and bio(macro)molecules, the immune response stimulated by glycoconjugates is negligible [5, 6], that is, high affinity carbohydrate-specific IgG-isotype antibodies are not easily obtained. Even if anticarbohydrate antibodies are generated, IgG comprises no more than 28% of the antibodies (74 IgGs in a total of 268 antibodies, with the remainder being IgMs) [7]. Therefore, while anticarbohydrate antibodies of the IgG isotype are preferred for carbohydrate research, IgM-antibodies with low affinity have been often used. Moreover, obtaining pure and homogeneous carbohydrates (or glycoconjugates) is very difficult. This is because regioselective protection of the hydroxy groups of the monosaccharide is required. Programmable one-pot oligosaccharide synthesis is widely performed using protected monosaccharides and/or oligosaccharides [8–10]. Enzyme-catalyzed oligosaccharide synthesis has been also developed [10–12]. Several oligosaccharides such as KH-1 antigen (nonasaccharide of Le^Y^-Le^X^), globo-H hexasaccharide, and the core pentamannosides have been prepared by automated solid-phase oligosaccharide synthesis [8]. However, due to the complicated procedures of carbohydrate preparation, a general methodology for their chemical synthesis is not yet established.

**Figure 1:**
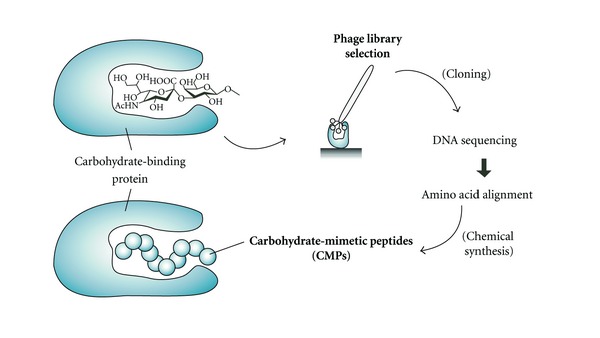
Identification of carbohydrate-mimetic peptides (CMPs) by affinity selection from a phage-display library. Selection is performed against carbohydrate-binding proteins. The peptides identified are chemically synthesized and recognized by the carbohydrate-binding protein.

To compensate for the lack of synthetic carbohydrates and to overcome their inherent weak immunogenicity, short peptides that bind to carbohydrate-binding proteins have been identified from phage-display libraries (Figure 1). These peptides mimic carbohydrate structures [13] and are called “carbohydrate-mimetic peptides (CMPs)” or “peptide mimotopes.” It is predicted that CMPs, as well as carbohydrates, are recognized by carbohydrate-binding proteins. Small molecules such as biotin and carbohydrate mimotope (Glycotope) mimicking peptides have been frequently identified, and a number of reviews focusing on different aspects of their properties and uses have been published [14–16]. In this paper, recent studies on the selection and application of CMPs are surveyed and summarized according to the classification of target carbohydrate-binding proteins.

## 2. Peptide Selection from Phage Display Libraries

**Figure 2:**
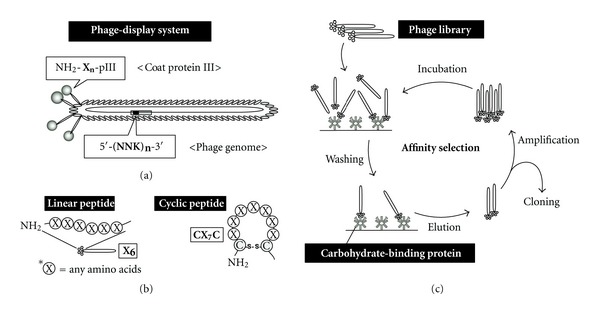
Phage-display system for affinity selection. (a) A typical filamentous phage carrying a peptide library. Foreign peptides (X_*n*_) are displayed on the N-terminus of coat protein III (pIII) (type 3; M13 or fd phage). An oligonucleotide coding peptide library [-(NNK)_*n*_-] is inserted into the phage genome. X = any amino acid; N = A, C, G, or T; K = G or T. (b) Linear (hexamer, *left*) and cyclic (heptamer, *right*) peptide libraries. (c) Schematic representation of the procedure for affinity selection (biopanning). The phage library is incubated with target receptors (carbohydrate-binding proteins), and unbound phages are removed by washing. Bound phages are eluted, amplified in *E. coli*, and subjected to the next cycle of biopanning. The cycle is repeated several times to enrich target-specific phages. Individual enriched phages are isolated and used for DNA sequencing.

Phage display is an efficient selection (and screening) system for the identification of target-specific peptides and proteins from a large number of candidates [20–22]. A filamentous virus (M13 and fd, etc.) that infects *E. coli* is frequently used in phage display technology. When DNA encoding foreign sequences is inserted into the coat protein (pIII or pVIII) region in the virus genome (M13 phage vector, etc.), the corresponding sequence is fused with the coat protein of the viral particle (Figure 2(a)) [20]. The foreign sequence is “displayed” on the viral particle and is able to interact with various types of target molecules.

In the case of peptide libraries, the length of the peptides is often 5–20 amino acids. There are two types of peptide library: linear peptide libraries and cyclic peptide libraries (Figure 2(b)). The randomized region of cyclic peptide libraries is surrounded by two cysteines (e.g., CX_7_C) to restrict the peptide conformation via disulfide bonds. The diversity of a peptide library is often 10^8^-10^9^, which is sufficient to cover a combination of hexapeptide libraries (X_6_; 20^6^ = 6.4 × 10^7^). Several kinds of peptide libraries (e.g., Ph.D. Phage Display Peptide Library Kits, New England Biolabs) and customizable phage vectors (Ph.D. Peptide Display Cloning System) are commercially available.

To isolate phage clones that have high affinity for a target molecule, a set of procedures called “affinity selection (biopanning)” is performed (Figure 2(c)). First, the target molecule is incubated with the phage library in order to bind to specific peptide sequences. After removal of excess phages by washing, the bound phages are eluted by incubation with a known ligand for the target or an acidic buffer. The phages are amplified by infection of hosts (*E. coli*), and the phage pool is subjected to another round of biopanning. By repeating these steps, target-binding phages are enriched, and, finally, phage clones are obtained. The peptides with high affinity for the target molecule are identified by DNA sequencing of individual phage clones. Huang and coworkers established a mimotope database MimoDB (http://immunet.cn/mimodb/) that contains the results of biopanning experiments including the phage libraries used and the peptide sequences identified [23, 24]. This database will help in the development of therapeutic molecules and the identification of superior peptide mimotopes for vaccination.

## 3. CMPs against Lectins

### 3.1. Monosaccharide-Mimetic Peptides

Most lectins recognize monosaccharides and disaccharides [4]. Concanavalin A (ConA) is a lectin from jack-bean (*Canavalia ensiformis*) that binds to *α*-mannose (*α*-Man) and *α*-glucose (*α*-Glc). ConA is a famous lectin that is commercially available for the biological investigation of glycoconjugates. The first CMPs were selected from a random peptide library against ConA simultaneously by Oldenburg et al. (octapeptide library) [25] and Scott et al. (hexapeptide library) [13] (Table 1). Peptides containing the consensus sequence, Tyr-Pro-Tyr (YPY), showed high affinity for ConA with a dissociation constant (*K*_d_) of 46 *μ*M, and the *K*_d_ for methyl *α*-Man was 89 *μ*M. The peptides are considered to mimic the structure of carbohydrates because the ConA-peptide interaction was inhibited by *α*-Man.

**Figure 3:**
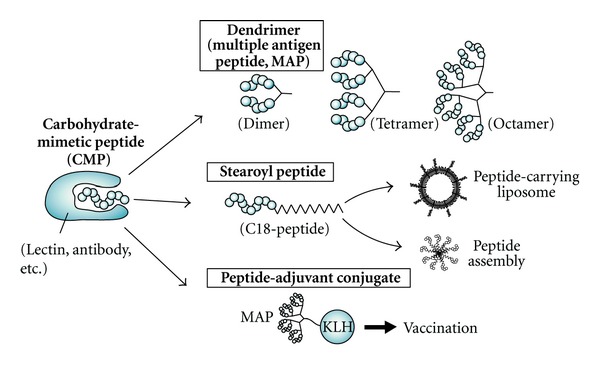
Representative chemical modifications of CMPs. To enhance the binding affinity, multiple CMPs are synthesized to give dimeric, tetrameric, and octameric dendrimers (multiple antigen peptide; MAP) (*upper*). The dendrimers are further conjugated with biotin, fluorescence groups, or adjuvants for vaccination. The peptide is modified with an alkyl group (stearic acid), enabling the peptide lipid to be incorporated into liposomes or to undergo self-assembly (*middle*). Monomeric CMP or CMP dendrimers are conjugated with adjuvants such as keyhole limpet hemocyanin (KLH), QS-21, and so forth (*lower*). The peptide-adjuvant conjugate is vaccinated into animals.

To obtain Man/Glc-mimetic peptides, Yu et al. used three lectins, including ConA, *Lens culinaris* agglutinin (LCA) from lentil, and *Pisum sativum* agglutinin (PSA) from pea [31]. Two cyclic peptides, CNTPLTSRC and CSRILTAAC, were selected from a cyclic heptapeptide library, but these peptides did not contain the YPY motif. Docking simulation of the peptide-lectin interaction suggested that the cyclic peptides bound to an alternative binding site, not to the sugar-binding site that is recognized by YPY-containing peptides. In another screen using monosaccharide-binding lectins, Eggink and Hoober identified a GalNAc/Gal-mimetic dodecapeptide, VQATQSNQHTPR, that was selected against *Helix pomatia* (HPA) lectin [32]. A tetrameric dendrimer of the peptide, [(VQATQSNQHTPR)_2 _K]_2 _K, was synthesized chemically (Figure 3), which was shown to stimulate the secretion of interleukin (IL)-8 and IL-21 from human peripheral blood mononuclear cells (PBMCs).

### 3.2. Disaccharide-Mimetic Peptides

**Figure 4:**
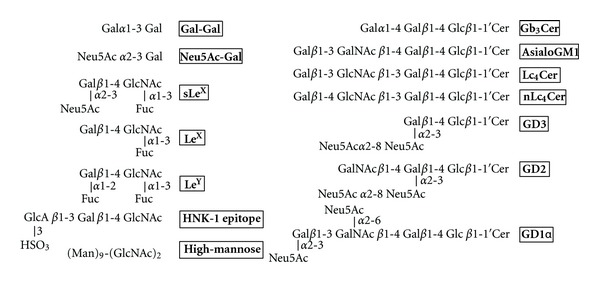
Oligosaccharide structures of carbohydrate antigens that are mimicked by peptides.

The Gal*α*1-3Gal disaccharide is recognized by *Griffonia simplicifolia* I-B4 (GS-I-B4) and *Bandeiraea simplicifolia* isolectin B4 (BS-I-B4) (Figure 4). The Gal*α*1-3Gal structure is a major carbohydrate antigen recognized by human anti-pig antibodies, and inhibitors of human natural antibodies may be useful in pig-to-human xenotransplantation. Kooyman et al. identified a peptide sequence, SSLRGF, that binds to GS-I-B4 lectin from a hexapeptide library [27]. Zhan et al. identified a peptide, NCVSPYWCEPLAPSARA, by selection with BS-I-B4 lectin [28]. These peptides, SSLRGF and NCVSPYWCEPLAPSARA, inhibited the agglutination of pig red blood cells (RBCs) by human serum. Two peptides, FHENWPS and FHEFWPT, that inhibit the agglutination of RBCs were identified by selection against anti-Gal antibody by Lang et al. [42]. However, the peptides identified from three selections contained no obvious consensus sequence.

**Figure 5:**
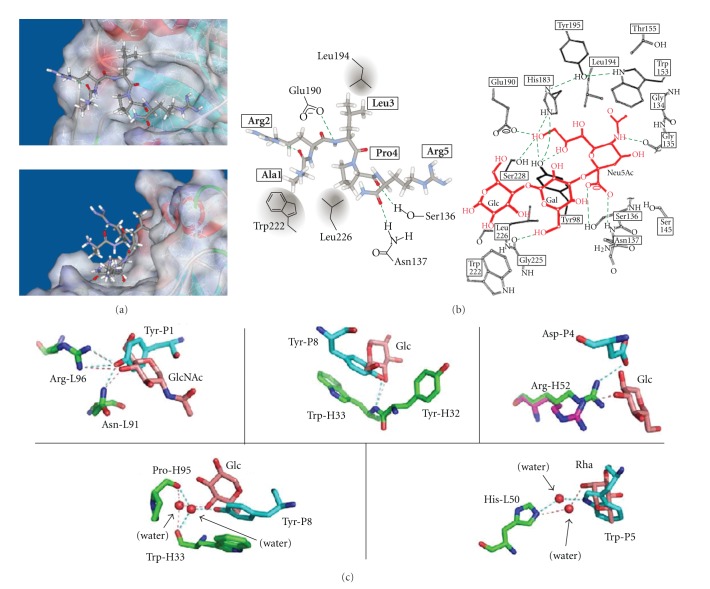
(a) Computer simulation of the interaction between peptide s2(1–5) and HA. A docking pose of the s2(1–5)-HA complex (*left*) and schematic diagram of the binding site of HA (*right*). The peptide is thought to be recognized by the Neu5Ac-Gal receptor-binding pocket. The peptide is shown as a stick model. Three potential hydrogen bonds (green dotted lines) between H3 and s2(1–5) are proposed (Glu190-Leu3, Ser136-Pro4, and Asn137-Arg5), which are similar to those in H3-Neu5Ac. Adapted from reference [17]. (b) Schematic diagram of the binding site of H3HA (Protein Data Bank entry, 1HGG). Neu5Ac*α*2–3Gal-Glc (sialyllactose) is shown in red. Modified from [18]. (c) Comparison of the polar interactions shown in the oligosaccharide (*O*-antigen of *S. flexneri* serotype 2a) and peptide B1 (YLEDWIKYNNQK) complexes of monoclonal antibody F22-4. The peptide and oligosaccharide ligands are distinguished by carbon atoms shown in cyan and pink, respectively (P, peptide; Rha, rhamnose). The carbon atoms of the F22-4 residues are shown in green (H, heavy chain; L, light chain). Adapted from [19].

Influenza virus hemagglutinin (HA) recognizes sialylgalactose structures (Neu5Ac-Gal) in glycoproteins and glycolipids on the cell surface in the initial stage of the infection process (Figure 4). Matsubara et al. identified CMPs from a pentadecapeptide library by selection with HAs of the H1 and H3 subtypes [17]. A HA-binding peptide, ARLSPTMVHPNGAQP, was identified from the first selection, and mutational sublibraries were prepared. A secondary selection was performed to improve the binding affinity for HAs, and the peptide was matured to peptide s2, ARLPRTMVHPKPAQP. The peptide was modified with a stearoyl group, and a molecular assembly of the alkylated peptides inhibited the infection of Madin-Darby canine kidney cells by influenza virus (Figure 3). Finally, a pentapeptide fragment from the N-terminal of s2, ARLPR [s2(1–5)], was found to show the highest inhibitory activity. A docking study of the interaction between the peptide s2(1–5) and HA suggested that the peptide is recognized by the Neu5Ac-Gal receptor-binding pocket (Figure 5(a)). The figure indicates that three side chains of H3HA (Ser 136, Asn137, and Glu190) have the potential to interact with the peptide instead of Neu5Ac, and hydrophobic residues (Leu194, Leu226, and Trp222) are close to the peptide (Figure 5(b)).

## 4. CMPs against Oligosaccharide-Binding Antibodies

### 4.1. Oligosaccharide-Mimetic Peptides for Inhibition

Glycoproteins and glycosphingolipids have unique oligosaccharide structures at their nonreducing termini [1]. Cell-cell communication is performed by oligosaccharides that are recognized by families of cell adhesion proteins such as selectins and sialic acid-binding immunoglobulin- (Ig-) like lectins (siglecs). Pathogenic viruses, toxins, and bacteria also recognize oligosaccharide structures [3]. Because an abundant variety of oligosaccharide structures relates to many carbohydrate-protein interactions, oligosaccharide-mimetic peptides mediate many kinds of inhibitory activities.

**Figure 6:**
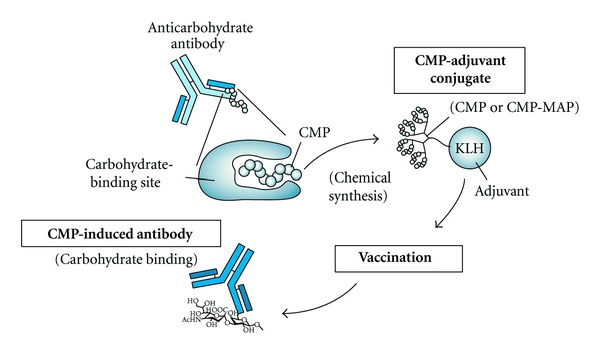
Procedure for obtaining CMP-induced antibodies by vaccination. A peptide mimotope (CMP) is conjugated with an adjuvant such as KLH and used for vaccination.

The sialyl-Lewis^X^ (sLe^X^) structure, Neu5Ac*α*2-3Gal*β*1-4(Fuc*α*1-3)GlcNAc, is recognized by E-selectin and is a famous carbohydrate antigen (Figure 4). sLe^X^-mimetic peptides were identified by selection against E-selectin [29, 30] and anti-sLe^X^ antibody [36] (Tables 1 and 2). Martens et al. identified the HITWDQLWNVMN peptide and further optimized the sequence as DITWDQLWDLMK using a mutagenesis library [29]. The binding affinity of the synthetic peptide for E-selectin was improved 100-fold by this optimization (IC_50_ for sLe^X^ binding to E-selectin; from 420 nM to 4 nM). The DITWDQLWDLMK peptide inhibited the adhesion of HL-60 cells and reduced neutrophil rolling on lipopolysaccharide- (LPS-) stimulated human umbilical vein endothelial cells. Qiu et al. designed WRY-containing peptides from the sLe^X^-mimetic peptide sequences, but these peptides cross-reacted with anti-Lewis^Y^ antibody. Octameric multiple antigen peptides (MAPs) were conjugated with QS-21 adjuvant, which resulted in cytotoxic IgM and IgG antibodies (Figures 3 and 6). MAPs, in which peptides are attached to an octabranched amino acid backbone, are used to generate antibodies against a synthetic peptide, which is useful for the design of vaccines [94]. Katagihallimath et al. selected a cyclic CSRLNYLHC peptide against anti-Le^X^ antibody [37]. The trisaccharide Le^X^ structure is known as CD15 or SSEA-1, and this structure is expressed in the developing and adult murine central nervous system. The Le^X^ mimetic peptide inhibited CD24-induced neurite outgrowth.

Neutral glycosphingolipid Lc_4_Cer-mimetic peptides showed unique activity [46] (Table 3). Lc_4_Cer contains Gal*β*1-3GlcNAc*β*1-3Gal*β*1-4Glc tetrasaccharide that is linked to ceramide (Figure 4), and Jack bean *β*-galactosidase digests a nonreducing terminus *β*-Gal to give Lc_3_Cer. The Lc_4_Cer-mimetic peptides inhibited digestion by *β*-galactosidase at a high concentration of enzyme, whereas the peptides enhanced the digestion of Lc_4_Cer at lower concentration of enzyme. This unique activity of the peptides was also shown in the digestion of nLc_4_Cer. This group also identified WHW-containing peptides such as WHWRHRIPLQLAAGR by selection with anti-GD1*α* antibody [47]. The ganglioside GD1*α* is cell adhesion molecule of murine metastatic large cell lymphoma (RAW117-H10 cells) that binds to endothelial cells. GD1*α*-mimetic peptides inhibited the adhesion between RAW117-H10 cells and hepatic sinusoidal endothelial (HSE) cells. Furthermore, the metastasis of RAW117-H10 cells to lung and spleen was completely inhibited by the intravenous injection of the peptide. Subsequently, WHW was found to be a minimal sequence that mimics the GD1*α* structure [48]. To modify the liposome surface with the WHW peptide, the WHW tripeptide was conjugated to alkyl groups such as palmitoyl or stearoyl groups (Figure 3). Coating of liposomes with peptides is often performed in drug delivery systems. The WHW-modified liposomes inhibited the adhesion between RAW117-H10 cells and HSE cells.

Tryptophan/tyrosine-containing tripeptides (YPY for ConA, WRY for sLe^X(Y)^, and WHW for GD1*α*) may comprise a key sequence that mimics oligosaccharide structure. Although Gb_3_ (Gal*α*1-4Gal*β*1-4Glc trisaccharide) is dissimilar to the disaccharide (Gal*β*1-3GlcNAc*β*) structure of Lc_4_ at the nonreducing terminus, Miura et al. identified a WHW-containing peptide (WHWTWLSEY) that mimics the Gb_3_ structure [60]. Gb_3_ is well known as a receptor for Shiga toxin (Stx). The Gb_3_-mimetic peptide showed neutralization activity against Stxs (Stx-1 and Stx-2) in a HeLa cell cytotoxicity assay. The binding affinity of the Gb_3_-mimetic peptide for Stx-1 was also investigated by surface plasmon resonance analysis (*K*_d_ = 1.4 nM).

### 4.2. Oligosaccharide-Mimetic Peptides for Vaccination

The immunogenicity of oligosaccharides is weak because oligosaccharides are ubiquitous components of cell membranes in tissues throughout the human body. When antioligosaccharide antibodies are generated, they attack these tissues and cause the risk of autoimmune disease. For example, lipopolysaccharides of *Campylobacter jejuni* isolated from GBS patients contain ganglioside-like epitopes such as GM1, GM1b, GD1a, and GalNAc-GDla, and these epitopes induce Guillain-Barre syndrome [95]. However, this low immunogenicity interferes with the preparation of antioligosaccharide antibodies that are useful for the investigation of glycoconjugate function.

To improve the binding affinity, specificity and cytotoxicity of antibodies, oligosaccharide-mimetic peptides are applied as peptide mimotopes of carbohydrate antigens for vaccination (Figure 6). Oligosaccharide-mimetic peptides were identified by selection against Le^X(Y)^ [34, 35, 37], sLe^X(Y)^ [36, 50], GD2 [36, 50–56], GD3 [36, 50, 59], lipooligosaccharide (LOS) [38, 39], *β*-1,2-oligomannoside [40], *N*-acetylglucosaminyl-*β*1,4-*N*-acetylmuramyl-alanyl-d-isoglutamine (GMDP) [43], and high-mannose oligosaccharide (Man_9_GlcNAc_2_ for HIV-1 gp120) [45]. The oligosaccharide-mimetic peptides were chemically synthesized and conjugated with adjuvant. To enhance the immunogenicity of the peptides, MAPs were prepared and resulted in dimeric, tetrameric, and octameric dendrimers (Figure 3). The peptide-adjuvant conjugates were vaccinated, with the adjuvants used being keyhole limpet hemocyanin (KLH) [39, 40, 53, 54], QS-21 [36, 50, 54], diphtheria toxoid (DT) [38], ovalbumin (OVA) [43], or very small size proteoliposomes (VSSP) [59] (Figure 6, Tables 2 and 3). In some cases, DNA vaccination was also performed [55, 56]. The CMP-induced antibodies are able to bind to peptide mimotopes and carbohydrate antigens.

## 5. CMPs against Polysaccharide-Binding Antibodies

Most polysaccharide-mimetic peptides to be applied for vaccination are identified as peptide mimotopes of carbohydrate antigens (Figure 6). Capsular polysaccharides of microorganisms are carbohydrate antigens, and it is known that these polysaccharides cause meningoencephalitis in immunocompromised patients, particularly those with AIDS (polysaccharide from *Cryptococcus neoformans*), pneumonia and bacteremia (*Streptococcus pneumoniae*), bacterial meningitis (*Neisseria meningitidis*), cholera (*Vibrio cholerae*), tuberculosis (*Mycobacterium tuberculosis*), and so forth (Table 4). These peptide mimotopes are potential antigens for safe vaccination and are expected to produce highly cytotoxic antibodies.

The typical methodology for vaccination uses a CMP-conjugated adjuvant. Valadon et al. identified CMPs that bind to anticryptococcal polysaccharide (glucuronoxylomannan, GXM) monoclonal antibody 2H1 [64]. The CMPs shared four motifs, for example, (E)TPXWM/LM/L and W/YXWM/LYE, and the dodecapeptide, GLQYTPSWMLVG (PA1) was found to bind 2H1 with a *K*_d_ of 295 nM [64]. The three-dimensional structure of 2H1 has been solved in a complex with PA1 [65]. The peptide PA1 was improved by selection from a PA1-based sublibrary, which identified the peptide P206-1 (FGGETFTPDWMMEVAIDNE) [66]. The affinity of peptide 206-1 for 2H1 was 80-fold higher than that of PA1 (*K*_d_ of 3.7 nM). Immunization of mice with P206-1-tetanus toxoid (TT), but not PA1 or P601E (DGASYSWMYEA), induced an anti-GXM response [66, 67].

Although antibodies against the capsular polysaccharide of the same species (e.g., *Neisseria meningitidis* serogroup B) were used, the CMPs identified were different and shared no consensus motif [73, 75–80] (Table 4). This may be due to the different antibodies used (HmenB3, 9-2-L3, 30H12, Seam3, or 13D9), different primary peptide libraries (CX_6_C, X_9_, CX_9_C, X_12_, or X_15_), or different selection conditions. Harris et al. also concluded that the CMPs identified by each antibody possessed distinct consensus motifs [72]. A variety of peptide-conjugating adjuvants such as KLH, TT, BSA, OVA, proteasome, and thyroglobulin have been used. In some cases, phage particles were directly used for vaccination [80, 86, 88], and a high level of the IgG_2a_ subtype in the response against CMPs was shown [80].

Theillet et al. clarified the structural mimicry of *O*-antigen oligosaccharide by CMPs [19].  Figure 5(c) shows a structural representation of the antibody-peptide complex in which the sugar chains were replaced by amino acids. Glc and GlcNAc were replaced by Tyr or Asp, and one or more hydrogen bonds are indicated. On the other hand, high-mannose oligosaccharide-mimic peptide (2G12-1 peptide) binds to a neighboring pocket of the oligosaccharide (Table 2) [45]. The binding site for the DVFYPYPYASGS peptide, which was selected against ConA, was different from the mannose/trimannose-binding site [26]. However, the peptide inhibits *α*-mannopyranoside binding to ConA [25], indicating that this peptide shows functional mimicry rather than structural mimicry.

## 6. Conclusion

Anticarbohydrate antibodies are necessary for clarifying the biological functions of carbohydrates, the detection of carbohydrates during etiological diagnosis, and therapy for carbohydrate-related diseases [7, 96]. Due to the difficulty in obtaining homogeneous glycoconjugates and carbohydrate-binding proteins, phage display libraries have been applied for the identification of peptide mimotopes. In this paper, the selection of CMPs was classified according to the types of target carbohydrates. The first selection was performed against lectins, and then the selections were performed against anticarbohydrate antibodies. To apply the peptide mimotopes for vaccination, this methodology is becoming more widespread.

## Figures and Tables

**Table 1:** Summary of the selection of CMPs with lectins.

Target lectins (abbreviations)	Peptide library	Peptide motif or representative sequences (peptide name)	Lectin-binding carbohydrate structures	References	Notes*
Concanavalin A (ConA)	X_8_, X_6_	YPY motif	Man; Glc	[13, 25, 26]	Inhibition of Man binding
*Griffonia simplicifolia* I-B4 isolectin (GS-I-B4)	X_6_	SSLRGF	Gal*α*1-3Gal	[27]	Inhibition of RBC agglutination
*Bandeiraea simplicifolia* I-B4 isolectin (BS-I-B4)	XCX_15_	NCVSPYWCEPLAPSARA	Gal*α*1-3Gal	[28]	Inhibition of RBC agglutination
E-selectin	X_12_	DITWDQLWDLMK	Sialyl Lewis^X^ [Neu5Ac*α*2-3Gal*β*1-4(Fuc*α*1–3)GlcNAc]	[29]	Inhibition of cell adhesion, reduction of neutrophil rolling, and so forth
	X_7_	IELLQAR		[30]	Octameric MAP, inhibition of HL-60, and B16 cell adhesion
Concanavalin A (ConA); *Lens culinaris* agglutinin (LCA); *Pisum sativum* agglutinin (PSA)	X_12_, CX_7_C	CNTPLTSRC; CSRILTAAC	Man; Glc	[31]	Inhibition of Man binding; docking study
Lectin from *Helix pomatia* (HPA)	X_12_	VQATQSNQHTPRGGGS	*O*-linked *α*-GalNAc; Gal*β*1-3GalNAc; *α*-GlcNAc	[32]	Tetrameric dendrimer, stimulation of IL-8, and IL-21 secretion
Lipopolysaccharide (LPS) binding protein (LBP); CD14	X_12_	FHRWPTWPLPSP (MP12)	Lipopolysaccharide	[33]	Inhibition of LPS-induced INF-*α* expression
Influenza virus hemagglutinin (HA)	X_15_	ARLPRTMVHPKPAQP (s2); ARLPR [s2(1–5)]	Neu5Ac*α*2-3Gal	[17]	*N*-stearoyl peptide; inhibition of flu infection

*RBC: red blood cell; IL: interleukin; INF: interferon.

**Table 2:** Summary of the selection of CMPs with oligosaccharide-binding antibodies.

Target antibodies (abbreviations)	Name of antibody	Peptide library	Peptide motif or representative sequences (peptide name)	Carbohydrate antigen	References	Notes*
Anti-Lewis^Y^ (Le^Y^)	B3	X_8_	APWLYGPA	Fuc*α*1-2Gal*β*1-4(Fuc*α*1-3)GlcNAc	[34, 35]	Induction of anti-Le^Y^ immune responses
	BR55-2; 15-6A	X_15_	WRY-containing peptide		[36]	Cross-reaction with anti-Le^X^
Anti-sialyl Lewis^X^ (sLe^X^)	FH-6	X_15_	WRY-containing peptide	Neu5Ac*α*2-3Gal*β*1-4(Fuc*α*1-3)GlcNAc	[36]	Cross-reaction with anti-Le^Y^; octameric MAP-QS21
Anti-Lewis^X^ (Le^X^)	L5	X_12_, CX_7_C	CSRLNYLHC	Gal*β*1-4(Fuc*α*1-3)GlcNAc	[37]	Inhibition of CD24-induced neurite outgrowth
Antilipooligosaccharide (LOS)	—	X_7_	SMYGSYN, APARQLP	LOS of group B *Neisseria meningitidis *	[38]	Peptide-DT
Antilipooligosaccharide (LOS)	—	X_12_	NMMRFTSQPPNN and so forth	LOS of nontypeable *Haemophilus influenzae *	[39]	Peptide-KLH
Anti-*β*-1,2-oligomannoside	DJ2.8	X_7_	FHENWPS	*β*-1,2-oligomannoside	[40]	Peptide-KLH
Anti-L2/HNK-1	L2-412	X_15_	FLHTRLFVSDWYHTP, FLHTRLFV	SO_4_-3GlcA*β*1-3Gal*β*1-4GlcNAc	[41]	Promotion of neurite outgrowth
Anti-Gal	B	X_7_, CX_7_C	FHENWPS, FHEFWPT	Xenoreactive *α*-Gal antigenic epitope	[42]	Inhibition of RBC agglutination
Anti-GMDP	E6/1.2	X_15_	RVPPRYHAKISPMVN	*N*-acetylglucosaminyl-*β*1,4-*N*-acetylmuramyl-alanyl-d-isoglutamine (GMDP)	[43]	Peptide-OVA
Antiglucitollysine	41; 226	X_12_, CX_9_C	CTSRXC motif	Glc-Lys	[44]	Inhibition of Glu-Lys binding
Antihigh-mannose oligosaccharides	2G12	X_15_CX	ACPPSHVLDMRSGTCLAAEGK (2G12.1)	Man_9_GlcNAc_2_ (HIV-1 gp120)	[45]	X-ray analysis (no structural mimic)

*DT: diphtheria toxoid; KLH: keyhole limpet hemocyanin; OVA: ovalbumin.

**Table 3:** Summary of the selection of CMPs with glycolipid-binding antibodies.

Target antibodies	Name of antibody	Peptide library	Peptide motif or representative sequences (peptide name)	Glycolipid structures	References	Notes*
Anti-Lc_4_Cer; anti-nLc_4_Cer	AD117m; H11	X_15_	VPPXFXXXY	Gal*β*1-3GlcNAc*β*1-3Gal*β*1-4Glc*β*1-1′Cer; Gal*β*1-4GlcNAc*β*1-3Gal*β*1-4Glc*β*1-1′Cer	[46]	Modulation of *β*-galactosidase activity
Anti-GD1*α*	KA17	X_15_	WHWRHRIPLQLAAGR	Neu5Ac*α*2-3Gal*β*1-3(Neu5Ac*α*2-6)GalNAc*β*1-4Gal*β*1-4Glc*β*1-1′Cer	[47, 48]	Inhibition of metastasis; peptide-liposome
Anti-asialo GM1	clone 10	X_7_, CX_7_C	KL/VWQXXX	Gal*β*1-3GalNAc*β*1-4Gal*β*1-4Glc*β*1-1′Cer	[49]	(Phage ELISA only)
Anti-GD3/GD2	ME36.1	X_15_	WRY-containing peptide and so forth	Neu5Ac*α*2-8Neu5Ac*α*2-3Gal*β*1-4Glc*β*1-1′Cer; GalNAc*β*1-4(Neu5Ac*α*2-8Neu5Ac*α*2-3)Gal*β*1-4Glc*β*1-1′Cer	[36, 50, 51]	Octameric MAP-QS21/KLH
Anti-GD2	14.18	CX_10_C	CDGGWLSKGSWC; CGRLKMVPDLEC	GalNAc*β*1-4(Neu5Ac*α*2-8Neu5Ac*α*2-3)Gal*β*1-4Glc*β*1-1′Cer	[52–54]	Docking study; peptide-KLH
	14G2a	X_15_	EDPSHSLGLDVALFM		[55, 56]	Molecular modeling; DNA vaccine; induction of CD8^+^ T cell response
	14G2a	XCX_8_CX	RCNPNMEPPRCF		[57, 58]	Inhibition of antibody binding to IMR-32 cells
Anti-GD3	4F6	X_15_	LAPPRPRSELVFLSV (GD3P4)	Neu5Ac*α*2-8Neu5Ac*α*2-3Gal*β*1-4Glc*β*1-1′Cer	[59]	Peptide-VSSP
Anti-Gb_3_Cer	—	X_12_	WHWTWLSEY	Gal*α*1-4Gal*β*1-4Glc*β*1-1′Cer	[60]	Neutralization of Shiga toxin
Anti-Neu5Gc-containing ganglioside (Neu5Gc-GM3)	1E10; chimeric P3; 1E10	X_9_, X_12_	KPPR, RRPR/K; LEICSYTPDEGC; KCGHHYCRQVDL	Neu5Gc*α*2-3Gal*β*1-4Glc*β*1-1′Cer	[61, 62]	Inhibition of 1E10 binding to P3
Anti-phenolic glycolipid-I (PGL-I)	III603.8	X_7_	W(T/R)LGPY(V/M)	*Mycobacterium leprae*-specific antigen	[63]	Does not bind to antibodies in serum

*MAP: multiple antigen peptide; VSSP: very small size proteoliposomes.

**Table 4:** Summary of the selection of CMPs with polysaccharide-binding antibodies.

Species	Carbohydrate antigen	Name of antibody	Peptide library	Peptide motif or representative sequences (peptide name)	References	Notes*
*Cryptococcus* *neoformans *	Polysaccharide (glucuronoxylomannan; GXM)	2H1	X_6_, X_10_, ADVA X_6_ TPXW [M/L][M/L] X_6_ AAG	(E)TPXWM/LM/L, W/YXWM/LY; GLQYTPSWMLVG (PA1); SYSWMYE (P601E); FGGETFTPDWMMEVAIDNE (P206.1)	[64–67]	Four motifs; X-ray analysis (PA1); peptide evolution (P206.1); peptide-KLH/TT
*Streptococcus* species	Capsular polysaccharide (type 3, group B)	S9	X_9_	WENWMMGNA; FDTGAFDPDWPA	[68]	Group B streptococci (GBS); peptide-KLH/BSA/OVA
*Streptococcus* *pneumoniae *	Capsular polysaccharide (serotype 4)	mAb4	X_15_	SGQARVLYSEFINAL (pep4)	[69]	DNA vaccine
	(serotype 8)	(human mAb IgA)	X_12_	FHLPYNHNWFAL (PUB1)	[70]	Peptide-TT
	(serotype 6B, 9V)	206; F-5; Db3G9	X_12_, X_15_	MP7, 12, 55, 58	[71]	Peptide-KLH
*Streptococcus pyogenes*	Cell-wall polysaccharide (group A)	SA-3; Strep9; HGAC39; HGAC47; HGAC101	X_6_, XCX_8_CX, X_8_CX_8_, X_15_CX, X_15_	DRPVPY	[72]	Basis of peptide-carbohydrate cross-reactivity
*Streptococcus agalactiae*	(serotype A, B, C)	(IgG2, Ig polyclonal)	X_12_	NPDHPRVPTFMA (2–8); LIPFHKHPHHRG (3-2)	[73]	DNA vaccine; MAP-CFA/IFA
*Neisseria meningitidis*	Capsular polysaccharide (serogroup A)	9C10	X_15_	GEASGLCCRWSSLKGC	[74]	Peptide-proteasome
	(serogroup B)	HmenB3	X_12_	NKVIWDRDWMYP	[75]	Peptide-BSA-CFA/IFA
	(serogroup B)	9-2-L3, 7, 9	CX_6_C, CX_9_C	CGAVIDDC	[76]	Peptide-KLH
	(serogroup B) [poly-*α*2–8 sialic acid (PSA)]	30H12	CX_9_C	CSSVTAWTTGCG	[77, 78]	Enhanced migration of grafted neuroblasts in mouse brain
	(serogroup B)	Seam3	X_9_, X_12_	DYAWDQTHQDPAK (9M)	[79]	Peptide-KLH, DNA vaccine
	(serogroup B)	13D9	X_15_	RGDKSRPPVWYVEGE	[80]	Phage vaccine
	(serogroups B and C)	(IgG2, Ig polyclonal)	X_12_	EQEIFTNITDRV (G3)	[73]	DNA vaccine; MAP-CFA/IFA
	(serogroup C)	1E4	X_15_	GFSYYRPPWIL (Pep2C)	[81]	Peptide-proteasome
	[*N*-propionyl derivative of CPS]	—	—	—	[82]	
*Vibrio cholerae*	Capsular polysaccharide (serogroup O139)	Vc1; Vc2; ICL12	six libraries (X_9_, X_12_, X_28_ etc.)	AEGEFSPGVWKAAFQGDKLPDPAK and so forth	[83]	Peptide-KLH/BSA
	(serogroup O1) Ogawa serotype	S-20-4; A-20-6	X_12_, X_7_, CX_7_C	NHNYPPLSLLTF (4P-8)	[84]	Peptide-KLH/BSA; docking study
	(serogroup O1) Ogawa and Inab serotypes	72.1	X_12_, X_7_, CX_7_C	ECLLLSKYCMPS (3ME-1); SMCMHGGAYCFP (3ME-2)	[85]	Peptide-KLH/BSA-CFA/IFA
*Shigella flexneri*	O-antigen of lipopolysaccharide (serotype 5a)	mIgAs C5; I3	X_9_	YKPLGALTH; KVPPWARTA	[86]	Phage vaccine
	(serotype 2a)	F22-4	X_12_	YLEDWIKYNNQK (B1)	[19]	X-ray analysis
—	Melanoma-associated chondroitin sulfate proteoglycan (MCSP)	763.74	X_6_	VHINAH	[87]	Inhibition of MCSP binding
*Entamoeba histolytica*	GPI-linked proteophosphoglycan antigens	EH5	six libraries (X_9_, X_12_, X_28_ etc.)	GTHPXL	[88]	Glycosylphosphatidylinositol (GPI); phage vaccine
*Mycobacterium tuberculosis*	Neutral polysaccharides	—	X_12_	QEPLMGTVPIRAGGGS (P1)	[89]	Peptide oligomer vaccine
*Mycobacterium tuberculosis*	Mannosylated lipoarabinomannan	CS40	X_12_	ISLTEWSMWYRH (B11)	[90]	Peptide-KLH-adjuvants
*Burkholderia pseudomallei*	Exopolysaccharide (EPS)	3VIE5; 4VA5	X_12_, X_7_, CX_7_C	CYLPFQLSC; CHPLFDARC	[91]	Peptide-thyroglobulin
*Brucella melitensis*; *Brucella abortus *	Lipopolysaccharide	4F9; 11B2	X_9_, X_12_, CX_9_CX, X_15_	WTEIHDWEAAME	[92]	DNA vaccine
*Staphylococcus aureus*	Peptidoglycan	—	X_12_	Sp-31	[93]	MAP vaccine

*BSA: bovine serum albumin; TT: tetanus toxoid; CFA: complete Freund's adjuvant; IFA: incomplete Freund's adjuvant.

## References

[1] Varki A (1993). Biological roles of oligosaccharides: all of the theories are correct. *Glycobiology*.

[2] Takahashi K, Tanabe K, Ohnuki M (2007). Induction of pluripotent stem cells from adult human fibroblasts by defined factors. *Cell*.

[3] Lis H, Sharon N (1998). Lectins: carbohydrate-specific proteins that mediate cellular recognition. *Chemical Reviews*.

[4] Weis WI, Drickamer K (1996). Structural basis of lectin-carbohydrate recognition. *Annual Review of Biochemistry*.

[5] Nores GA, Lardone RD, Comín R, Alaniz ME, Moyano AL, Irazoqui FJ (2008). Anti-GM1 antibodies as a model of the immune response to self-glycans. *Biochimica et Biophysica Acta*.

[6] Slovin SF, Keding SJ, Ragupathi G (2005). Carbohydrate vaccines as immunotherapy for cancer. *Immunology and Cell Biology*.

[7] Kannagi R, Hakomori S (2001). A guide to monoclonal antibodies directed to glycotopes. *Advances in Experimental Medicine and Biology*.

[8] Hsu C-H, Hung S-C, Wu C-Y, Wong C-H (2011). Toward automated oligosaccharide synthesis. *Angewandte Chemie*.

[9] Boltje TJ, Buskas T, Boons GJ (2009). Opportunities and challenges in synthetic oligosaccharide and glycoconjugate research. *Nature Chemistry*.

[10] Nishimura SI (2001). Combinatorial syntheses of sugar derivatives. *Current Opinion in Chemical Biology*.

[11] Hancock SM, Vaughan MD, Withers SG (2006). Engineering of glycosidases and glycosyltransferases. *Current Opinion in Chemical Biology*.

[12] Daines AM, Maltman BA, Flitsch SL (2004). Synthesis and modifications of carbohydrates, using biotransformations. *Current Opinion in Chemical Biology*.

[13] Scott JK, Loganathan D, Easley RB, Gong X, Goldstein IJ (1992). A family of concanavalin A-binding peptides from a hexapeptide epitope library. *Proceedings of the National Academy of Sciences of the United States of America*.

[14] Fukuda MN (2012). Peptide-displaying phage technology in glycobiology. *Glycobiology*.

[15] Dudak FC, Boyaci IH, Orner BP (2011). The discovery of small-molecule mimicking peptides through phage display. *Molecules*.

[16] Cortese R, Felici F, Galfre G, Luzzago A, Monaci P, Nicosia A (1994). Epitope discovery using peptide libraries displayed on phage. *Trends in Biotechnology*.

[17] Matsubara T, Onishi A, Saito T (2010). Sialic acid-mimic peptides as hemagglutinin inhibitors for anti-influenza therapy. *Journal of Medicinal Chemistry*.

[18] Sauter NK, Hanson JE, Glick GD (1992). Binding of influenza virus hemagglutinin to analogs of its cell-surface receptor, sialic acid: analysis by proton nuclear magnetic resonance spectroscopy and X-ray crystallography. *Biochemistry*.

[19] Theillet FX, Saul FA, Vulliez-Le Normand B (2009). Structural mimicry of O-antigen by a peptide revealed in a complex with an antibody raised against *Shigella flexneri* serotype 2a. *Journal of Molecular Biology*.

[20] Smith GP, Petrenko VA (1997). Phage display. *Chemical Reviews*.

[21] Smith GP (1985). Filamentous fusion phage: novel expression vectors that display cloned antigens on the virion surface. *Science*.

[22] Scott JK, Smith GP (1990). Searching for peptide ligands with an epitope library. *Science*.

[23] Ru B, Huang J, Dai P (2010). MimoDB: a new repository for mimotope data derived from phage display technology. *Molecules*.

[24] Huang J, Ru B, Zhu P (2012). MimoDB 2.0: a mimotope database and beyond. *Nucleic Acids Research*.

[25] Oldenburg KR, Loganathan D, Goldstein IJ, Schultz PG, Gallop MA (1992). Peptide ligands for a sugar-binding protein isolated from a random peptide library. *Proceedings of the National Academy of Sciences of the United States of America*.

[26] Jain D, Kaur K, Sundaravadivel B, Salunke DM (2000). Structural and functional consequences of peptide-carbohydrate mimicry: crystal structure of a carbohydrate-mimicking peptide bound to concanavalin A. *Journal of Biological Chemistry*.

[27] Kooyman DL, Mcclellan SB, Parker W (1996). Identification and characterization of a galactosyl peptide mimetic. Implications for use in removing xenoreactive anti-a gal antibodies. *Transplantation*.

[28] Zhan J, Xia Z, Xu L, Yan Z, Wang K (2003). A peptide mimetic of Gal-*α*1,3-Gal is able to block human natural antibodies. *Biochemical and Biophysical Research Communications*.

[29] Martens CL, Cwirla SE, Lee RYW (1995). Peptides which bind to E-selectin and block neutrophil adhesion. *Journal of Biological Chemistry*.

[30] Fukuda MN, Ohyama C, Lowitz K (2000). A peptide mimic of E-selectin ligand inhibits sialyl Lewis X-dependent lung colonization of tumor cells. *Cancer Research*.

[31] Yu L, Yu PS, Yee Yen Mui E (2009). Phage display screening against a set of targets to establish peptide-based sugar mimetics and molecular docking to predict binding site. *Bioorganic and Medicinal Chemistry*.

[32] Eggink LL, Hoober JK (2009). A biologically active peptide mimetic of N-acetylgalactosamine/galactose. *BMC Research Notes*.

[33] Xu Z, Qian GS, Li Q, Feng QJ, Wu GM, Li KL (2009). Screening of mimetic peptides for CD14 binding site with LBP and antiendotoxin activity of mimetic peptide in vivo and in vitro. *Inflammation Research*.

[34] Hoess R, Brinkmann U, Handel T, Pastan I (1993). Identification of a peptide which binds to the carbohydrate-specific monoclonal antibody B3. *Gene*.

[35] Lou Q, Pastan I (1999). A Lewis(y) epitope mimicking peptide induces anti-Lewis(y) immune responses in rabbits and mice. *Journal of Peptide Research*.

[36] Qiu J, Luo P, Wasmund K, Steplewski Z, Kieber-Emmons T (1999). Towards the development of peptide mimotopes of carbohydrate antigens as cancer vaccines. *Hybridoma*.

[37] Katagihallimath N, Mehanna A, Guseva D, Kleene R, Schachner M (2010). Identification and validation of a Lewisx glycomimetic peptide. *European Journal of Cell Biology*.

[38] Charalambous BM, Feavers IM (2000). Peptide mimics elicit antibody responses against the outer-membrane lipooligosaccharide of group B *Neisseria meningitidis*. *FEMS Microbiology Letters*.

[39] Hou Y, Gu XX (2003). Development of peptide mimotopes of lipooligosaccharide from nontypeable Haemophilus influenzae as vaccine candidates. *Journal of Immunology*.

[40] Jouault T, Fradin C, Dzierszinski F (2001). Peptides that mimic Candida albicans-derived *β*-1,2-linked mannosides. *Glycobiology*.

[41] Simon-Haldi M, Mantei N, Franke J, Voshol H, Schachner M (2002). Identification of a peptide mimic of the L2/HNK-1 carbohydrate epitope. *Journal of Neurochemistry*.

[42] Lang J, Zhan J, Xu L, Yan Z (2006). Identification of peptide mimetics of xenoreactive *α*-Gal antigenic epitope by phage display. *Biochemical and Biophysical Research Communications*.

[43] Laman AG, Shepelyakovskaya AO, Berezin IA (2007). Identification of pentadecapeptide mimicking muramyl peptide. *Vaccine*.

[44] Rojas G, Pupo A, Del Rosario Aleman M, Santiago Vispo N (2008). Preferential selection of Cys-constrained peptides from a random phage-displayed library by anti-glucitollysine antibodies. *Journal of Peptide Science*.

[45] Menendez A, Calarese DA, Stanfield RL (2008). A peptide inhibitor of HIV-1 neutralizing antibody 2G12 is not a structural mimic of the natural carbohydrate epitope on gp120. *FASEB Journal*.

[46] Taki T, Ishikawa D, Hamasaki H, Handa S (1997). Preparation of peptides which mimic glycosphingolipids by using phage peptide library and their modulation on *β*-galactosidase activity. *FEBS Letters*.

[47] Ishikawa D, Kikkawa H, Ogino K, Hirabayashi Y, Oku N, Taki T (1998). GD1*α*-replica peptides functionally mimic GD1*α*, an adhesion molecule of metastatic tumor cells, and suppress the tumor metastasis. *FEBS Letters*.

[48] Takikawa M, Kikkawa H, Asai T (2000). Suppression of GD1*α* ganglioside-mediated tumor metastasis by liposomalized WHW-peptide. *FEBS Letters*.

[49] Qiu JX, Marcus DM (1999). Use of peptide ligands to analyze the fine specificity of antibodies against asialo GM1. *Journal of Neuroimmunology*.

[50] Kieber-Emmons T, Luo P, Qiu J, Chang TY, Blaszczyk-Thurin M, Steplewski Z (1999). Vaccination with carbohydrate peptide mimotopes promotes anti-tumor responses. *Nature Biotechnology*.

[51] Wondimu A, Zhang T, Kieber-Emmons T (2008). Peptides mimicking GD2 ganglioside elicit cellular, humoral and tumor-protective immune responses in mice. *Cancer Immunology, Immunotherapy*.

[52] Förster-Waldl E, Riemer AB, Dehof AK (2005). Isolation and structural analysis of peptide mimotopes for the disialoganglioside GD2, a neuroblastoma tumor antigen. *Molecular Immunology*.

[53] Riemer AB, Förster-Waldl E, Brämswig KH (2006). Induction of IgG antibodies against the GD2 carbohydrate tumor antigen by vaccination with peptide mimotopes. *European Journal of Immunology*.

[54] Bleeke M, Fest S, Huebener N (2009). Systematic amino acid substitutions improved efficiency of GD2-peptide mimotope vaccination against neuroblastoma. *European Journal of Cancer*.

[55] Bolesta E, Kowalczyk A, Wierzbicki A (2005). DNA vaccine expressing the mimotope of GD2 ganglioside induces protective GD2 cross-reactive antibody responses. *Cancer Research*.

[56] Wierzbicki A, Gil M, Ciesielski M (2008). Immunization with a mimotope of GD2 ganglioside induces CD8+ T cells that recognize cell adhesion molecules on tumor cells. *Journal of Immunology*.

[57] Horwacik I, Czaplicki D, Talarek K (2007). Selection of novel peptide mimics of the GD2 ganglioside from a constrained phage-displayed peptide library. *International Journal of Molecular Medicine*.

[58] Horwacik I, Kurciński M, Bzowska M (2011). Analysis and optimization of interactions between peptides mimicking the GD2 ganglioside and the monoclonal antibody 14G2a. *International Journal of Molecular Medicine*.

[59] Popa I, Ishikawa D, Tanaka M, Ogino K, Portoukalian J, Taki T (2006). GD3-replica peptides selected from a phage peptide library induce a GD3 ganglioside antibody response. *FEBS Letters*.

[60] Miura Y, Sakaki A, Kamihira M, Iijima S, Kobayashi K (2006). A globotriaosylceramide (Gb3Cer) mimic peptide isolated from phage display library expressed strong neutralization to Shiga toxins. *Biochimica et Biophysica Acta*.

[61] Perez A, Mier ES, Vispo NS, Vazquez AM, Rodríguez RP (2002). A monoclonal antibody against NeuGc-containing gangliosides contains a regulatory idiotope involved in the interaction with B and T cells. *Molecular Immunology*.

[62] López-Requena A, De Acosta CM, Moreno E (2007). Gangliosides, Ab1 and Ab2 antibodies. I. Towards a molecular dissection of an idiotype-anti-idiotype system. *Molecular Immunology*.

[63] Youn JH, Myung HJ, Liav A (2004). Production and characterization of peptide mimotopes of phenolic glycolipid-I of *Mycobacterium leprae*. *FEMS Immunology and Medical Microbiology*.

[64] Valadon P, Nussbaum G, Boyd LF, Margulies DH, Scharff MD (1996). Peptide libraries define the fine specificity of anti-polysaccharide antibodies to *Cryptococcus neoformans*. *Journal of Molecular Biology*.

[65] Young ACM, Valadon P, Casadevall A, Scharff MD, Sacchettini JC (1997). The three-dimensional structures of a polysaccharide binding antibody to *Cryptococcus neoformans* and its complex with a peptide from a phage display library: implications for the identification of peptide mimotopes. *Journal of Molecular Biology*.

[66] Beenhouwer DO, May RJ, Valadon P, Scharff MD (2002). High affinity mimotope of the polysaccharide capsule of *Cryptococcus neoformans* identified from an evolutionary phage peptide library. *Journal of Immunology*.

[67] Valadon P, Nussbaum G, Oh J, Scharff MD (1998). Aspects of antigen mimicry revealed by immunization with a peptide mimetic of *Cryptococcus neoformans* polysaccharide. *Journal of Immunology*.

[68] Pincus SH, Smith MJ, Jennings HJ, Burritt JB, Glee PM (1998). Peptides that mimic the group B streptococcal type III capsular polysaccharide antigen. *Journal of Immunology*.

[69] Lesinski GB, Smithson SL, Srivastava N, Chen D, Widera G, Westerink MAJ (2001). A DNA vaccine encoding a peptide mimic of *Streptococcus pneumoniae* serotype 4 capsular polysaccharide induces specific anti-carbohydrate antibodies in Balb/c mice. *Vaccine*.

[70] Buchwald UK, Lees A, Steinitz M, Pirofski LA (2005). A peptide mimotope of type 8 pneumococcal capsular polysaccharide induces a protective immune response in mice. *Infection and Immunity*.

[71] Smith CM, Passo CL, Scuderi A (2009). Peptide mimics of two pneumococcal capsular polysaccharide serotypes (6B and 9V) protect mice from a lethal challenge with *Streptococcus pneumoniae*. *European Journal of Immunology*.

[72] Harris SL, Craig L, Mehroke JS (1997). Exploring the basis of peptide-carbohydrate crossreactivity: evidence for discrimination by peptides between closely related anti-carbohydrate antibodies. *Proceedings of the National Academy of Sciences of the United States of America*.

[73] Wu Y, Zhang Q, Sales D, Bianco AE, Craig A (2010). Vaccination with peptide mimotopes produces antibodies recognizing bacterial capsular polysaccharides. *Vaccine*.

[74] Grothaus MC, Srivastava N, Smithson SL (2000). Selection of an immunogenic peptide mimic of the capsular polysaccharide of *Neisseria meningitidis* serogroup A using a peptide display library. *Vaccine*.

[75] Park I, Choi IH, Kim SJ, Shin JS (2004). Peptide mimotopes of *Neisseria meningitidis* group B capsular polysaccharide. *Yonsei Medical Journal*.

[76] Lauvrak V, Berntzen G, Heggelund U (2004). Selection and characterization of cyclic peptides that bind to a monoclonal antibody against meningococcal L3,7,9 lipopolysaccharides. *Scandinavian Journal of Immunology*.

[77] Torregrossa P, Buhl L, Bancila M (2004). Selection of poly-*α* 2,8-sialic acid mimotopes from a random phage peptide library and analysis of their bioactivity. *Journal of Biological Chemistry*.

[78] Marino P, Norreel JC, Schachner M, Rougon G, Amoureux MC (2009). A polysialic acid mimetic peptide promotes functional recovery in a mouse model of spinal cord injury. *Experimental Neurology*.

[79] Lo Passo C, Romeo A, Pernice I (2007). Peptide mimics of the group B meningococcal capsule induce bactericidal and protective antibodies after immunization. *Journal of Immunology*.

[80] Menéndez T, Santiago-Vispo NF, Cruz-Leal Y (2011). Identification and characterization of phage-displayed peptide mimetics of *Neisseria meningitidis* serogroup B capsular polysaccharide. *International Journal of Medical Microbiology*.

[81] Prinz DM, Smithson SL, Westerink MAJ (2004). Two different methods result in the selection of peptides that induce a protective antibody response to *Neisseria meningitidis* serogroup C. *Journal of Immunological Methods*.

[82] Moe GR, Granoff DM (2001). Molecular mimetics of *Neisseria meningitidis* serogroup B polysaccharide. *International Reviews of Immunology*.

[83] Falklind-Jerkérus S, Felici F, Cavalieri C (2005). Peptides mimicking *Vibrio cholerae* O139 capsular polysaccharide elicit protective antibody response. *Microbes and Infection*.

[84] Dharmasena MN, Jewell DA, Taylor RK (2007). Development of peptide mimics of a protective epitope of *Vibrio cholerae* Ogawa O-antigen and investigation of the structural basis of peptide mimicry. *Journal of Biological Chemistry*.

[85] Dharmasena MN, Krebs SJ, Taylor RK (2009). Characterization of a novel protective monoclonal antibody that recognizes an epitope common to *Vibrio cholerae* Ogawa and Inaba serotypes. *Microbiology*.

[86] Phalipon A, Folgori A, Arondel J (1997). Induction of anti-carbohydrate antibodies by phage library-selected peptide mimics. *European Journal of Immunology*.

[87] Geiser M, Schultz D, Le Cardinal A, Voshol H, García-Echeverría C (1999). Identification of the human melanoma-associated chondroitin sulfate proteoglycan antigen epitope recognized by the antitumor monoclonal antibody 763.74 from a peptide phage library. *Cancer Research*.

[88] Melzer H, Fortugno P, Mansouri E (2002). Antigenicity and immunogenicity of phage library-selected peptide mimics of the major surface proteophosphoglycan antigens of *Entamoeba histolytica*. *Parasite Immunology*.

[89] Gevorkian G, Segura E, Acero G (2005). Peptide mimotopes of *Mycobacterium tubercolosis* carbohydrate immunodeterminants. *Biochemical Journal*.

[90] Barenholz A, Hovav AH, Fishman Y, Rahav G, Gershoni JM, Bercovier H (2007). A peptide mimetic of the mycobacterial mannosylated lipoarabinomannan: characterization and potential applications. *Journal of Medical Microbiology*.

[91] Legutki JB, Nelson M, Titball R, Galloway DR, Mateczun A, Baillie LW (2007). Analysis of peptide mimotopes of *Burkholderia pseudomallei* exopolysaccharide. *Vaccine*.

[92] Beninati C, Garibaldi M, Passo CL (2009). Immunogenic mimics of *Brucella* lipopolysaccharide epitopes. *Peptides*.

[93] Chen Y, Liu B, Yang D (2011). Peptide mimics of peptidoglycan are vaccine candidates and protect mice from infection with *Staphylococcus aureus*. *Journal of Medical Microbiology*.

[94] Tam JP (1988). Synthetic peptide vaccine design: synthesis and properties of a high-density multiple antigenic peptide system. *Proceedings of the National Academy of Sciences of the United States of America*.

[95] Yuki N (2001). Infectious origins of, and molecular mimicry in, Guillain-Barré and Fisher syndromes. *Lancet Infectious Diseases*.

[96] Feizi T (1985). Demonstration by monoclonal antibodies that carbohydrate structures of glycoproteins and glycolipids are onco-developmental antigens. *Nature*.

